# Hybrid Sterility Locus on Chromosome X Controls Meiotic Recombination Rate in Mouse

**DOI:** 10.1371/journal.pgen.1005906

**Published:** 2016-04-22

**Authors:** Maria Balcova, Barbora Faltusova, Vaclav Gergelits, Tanmoy Bhattacharyya, Ondrej Mihola, Zdenek Trachtulec, Corinna Knopf, Vladana Fotopulosova, Irena Chvatalova, Sona Gregorova, Jiri Forejt

**Affiliations:** 1 Laboratory of Mouse Molecular Genetics, Division BIOCEV, Institute of Molecular Genetics, Academy of Sciences of the Czech Republic, Prague, Czech Republic; 2 Laboratory of Germ Cell Development, Division BIOCEV, Institute of Molecular Genetics, Academy of Sciences of the Czech Republic, Prague, Czech Republic; National Institutes of Health, UNITED STATES

## Abstract

Meiotic recombination safeguards proper segregation of homologous chromosomes into gametes, affects genetic variation within species, and contributes to meiotic chromosome recognition, pairing and synapsis. The *Prdm9* gene has a dual role, it controls meiotic recombination by determining the genomic position of crossover hotspots and, in infertile hybrids of house mouse subspecies *Mus m*. *musculus* (*Mmm*) and *Mus m*. *domesticus* (*Mmd*), it further functions as the major hybrid sterility gene. In the latter role *Prdm9* interacts with the hybrid sterility X 2 (*Hstx2)* genomic locus on Chromosome X (Chr X) by a still unknown mechanism. Here we investigated the meiotic recombination rate at the genome-wide level and its possible relation to hybrid sterility. Using immunofluorescence microscopy we quantified the foci of MLH1 DNA mismatch repair protein, the cytological counterparts of reciprocal crossovers, in a panel of inter-subspecific chromosome substitution strains. Two autosomes, Chr 7 and Chr 11, significantly modified the meiotic recombination rate, yet the strongest modifier, designated meiotic recombination 1, *Meir1*, emerged in the 4.7 Mb *Hstx2* genomic locus on Chr X. The male-limited transgressive effect of *Meir1* on recombination rate parallels the male-limited transgressive role of *Hstx2* in hybrid male sterility. Thus, both genetic factors, the *Prdm9* gene and the *Hstx2/Meir1* genomic locus, indicate a link between meiotic recombination and hybrid sterility. A strong female-specific modifier of meiotic recombination rate with the effect opposite to *Meir1* was localized on Chr X, distally to *Meir1*. Mapping *Meir1* to a narrow candidate interval on Chr X is an important first step towards positional cloning of the respective gene(s) responsible for variation in the global recombination rate between closely related mouse subspecies.

## Author Summary

During differentiation of germ cells into gametes, a maternal and a paternal copy of each chromosome have to find each other, pair, and synapse in order to ensure proper chromosome segregation into the gametes. Because of the unique ability to identify homologous DNA sequences between homologous chromosomes, meiotic recombination is an essential step in proper chromosome pairing and synapsis in the majority of species. However, when the paternal and maternal sets of chromosomes come from different (sub)species, the recognition of homologs can be disturbed and result in sterility of male hybrids. In this study we investigated the genetic control of variation in the global recombination rate between two closely related mouse subspecies with regard to the known infertility of their F1 hybrids. We show that the variation in the global recombination rate between both subspecies is under the control of three genomic loci. The strongest one appeared within the hybrid sterility X2 genomic locus on Chromosome X. Our findings will allow positional cloning of the gene and will shed new light on the role of meiotic recombination in reproductive isolation between closely related species.

## Introduction

Meiotic recombination of homologous chromosomes enhances genetic diversity of species and safeguards proper segregation of chromosomes into gametes. In the mouse the process begins at the leptotene stage of the first meiotic prophase with chromatin modification by PRDM9-directed trimethylation at lysine-4 of histone H3. Of approximately 4700 PRDM9-modified, nucleosome-depleted sites present in an average meiosis [1], ~250 are targeted by the SPO11 protein to induce programmed DNA double-strand breaks (DSBs) detectable by immunofluorescence as RAD51/DMC1 foci [2–4]. The foci represent single-stranded 3' DNA intermediates generated by 5'-strand resection of DSBs and bound by RAD51 and DMC1 strand exchange proteins. The resulting nucleoprotein filaments invade nearby DNA molecules in search of homologous DNA sequences and initiate synapsis of homologous chromosomes (but see [5]). In mice, approximately 90% of these DSBs are repaired by non-reciprocal recombination (NCO) and about 10% convert to reciprocal crossovers (COs), which can be traced in meiotic spreads as the MLH1 foci at mid- and late pachytene or as chiasmata at diplotene—metaphase I [6].

Meiotic COs are regulated at several levels. At the DNA sequence small scale, the distribution of DSBs and COs is highly nonrandom. The majority of DNA breaks occur in a subset of approximately 15 000 hotspots defined as 1 to 2kb long genomic intervals, with dramatically enhanced cM/DNA length ratio. The chance of a DSB to arise in a particular hotspot varies between 10–0.01% in a given cell, but is dramatically lower or zero outside the hotspots [7]. The nonrandom localization of hotspots is almost exclusively determined by the sequence-specific DNA binding of the zinc-finger array of PRDM9 meiosis-specific protein [8–11]. In *Prdm9*-null mutants the number of activated H3K4me3 hotspots remains constant but they move towards gene promoters and to other PRDM9-independent H3K4me3 sites [12], and probably cause male and female infertility [13]. The regulation of meiotic recombination also operates at the chromosome level, since at least one CO site has to occur per chromosome pair to secure proper synapsis and segregation of homologous chromosomes ("obligatory CO") [14]. Location of any additional CO on the same chromosome is constrained by positive interference [14–16]. The third and the least understood level of regulation operates at the genome-wide frequency of meiotic recombination, designated as the recombination/CO rate. The CO rate is under the genetic control in all properly analyzed species. More than 40 years ago we reported significant differences of male meiotic chiasma frequency between A/Ph, C57BL/10ScSn (high) and C3H/Di (low) inbred strains and suggested genetic control of CO rate in mice [17,18]. Recently, a variation in the global recombination rate has been reported in whole-genome F2 genetic linkage maps in the mouse [19], in a panel of human two-generation families [20] and in a large dairy cattle pedigree [21]. Detailed information on quantitative trait loci (QTL) controlling the meiotic CO rate in mice was obtained by counting autosomal MLH1 foci, the cytological counterparts of meiotic COs [22]. The counts varied between 20 and 30 foci per single meiosis in male mice, corresponding to 1000 cM and 1500 cM of the genome size on genetic maps [3,23–26].

It is important to note that besides specifying the position of recombination hotspots, *Prdm9* functions as a hybrid sterility gene in mouse intersubspecific PWD/Ph x C57BL/6J F1 hybrids. PWD/Ph (henceforth PWD) and C57BL/6J F1 (B6) inbred strains represent *Mus musculus musculus* (*Mmm*) and *Mus musculus domesticus* (*Mmd*) subspecies of the house mouse [27]. Sterility of male hybrids is controlled by the interaction between *Prdm9* and the X-linked Hybrid sterility X chromosome 2 locus, *Hstx2*, another major hybrid sterility factor [28]. The incompatibility between both hybrid sterility genes results in abnormal synapsis of homologous chromosomes, failure of sex body formation and consequent sterility of male F1 hybrids; all other allelic combinations yield fertile or semifertile phenotypes [29,30]. One of the probable explanations of disrupted synapsis between homologs in the first meiotic prophase could be the failure of proper repair of programmed DSBs caused by Dobzhansky-Muller incompatibility between *Prdm9* and *Hstx2*. Thus *Hstx2* might at some level participate in genetic control of meiotic recombination. This line of thought was inspired by the finding of an X-linked transgressive quantitative trait locus (QTL) controlling the global meiotic CO rate in crosses of another mouse subspecies, *Mus m*. *castaneus* (*Mmc*), with *Mmm*. The QTL was 18.8 Mb long, overlapping the 4.7 Mb long *Hstx2* genomic locus [24].

To inquire into the effect of PWD X-chromosome and individual PWD autosomes (Chrs) or their parts on global CO rate in the context of B6 genome [31] we estimated the average counts of MLH1 foci per pachynema in a panel of 26 chromosome substitution (consomic) strains C57BL/6-Chr#^PWD/Ph^/ForeJ (hereafter B6.PWD-Chr#) [32]. Chromosome substitution strains are generated by transfer of an entire chromosome from the donor inbred strain, in our case PWD, into the genetic background of the recipient strain (B6) by repeated backcrosses and selection at each backcross generation for a nonrecombinant donor chromosome [33].

In agreement with our prediction, the strongest, male-specific modifier was mapped on the X chromosome into the 4.7 Mb *Hstx2* genomic locus. In female meiosis, a strong modifier of global CO rate mapped also on Chr X, but distally to *Hstx2*. Two PWD autosomes also displayed a significant impact on the meiotic recombination rate. We further examined the meiotic CO rate in *Prdm9* deficient males and in males with *Prdm9* transgenes in order to compare the effect of *Prdm9* gene dosage on meiotic recombination and hybrid sterility.

## Results

**Fig 1:**
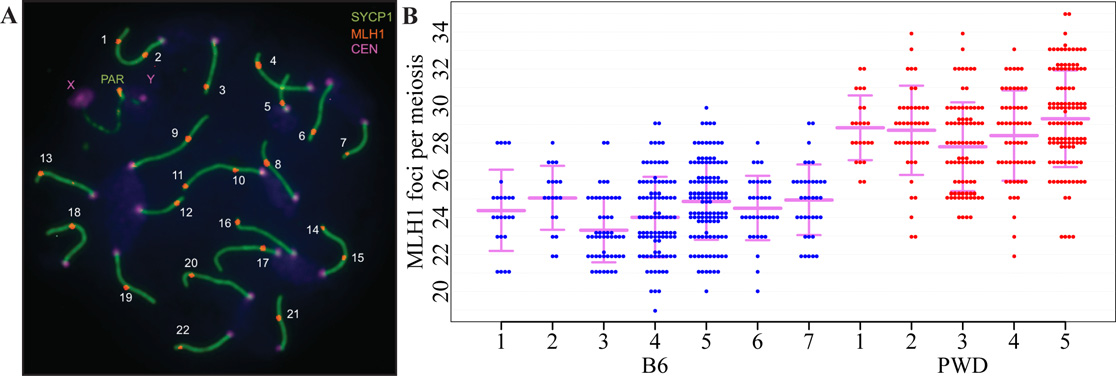
Variation in recombination rate of B6 and PWD male mice. (**A**) Pachytene spread of a B6 male meiosis shows central elements of synaptonemal complexes of 19 autosomes immunostained for SYCP1 (green), 22 MLH1 foci (red) and foci of centromeric proteins (violet). The number of MLH1 foci per nucleus was used as an equivalent of number of crossovers. The pseudoautosomal region (PAR) of Chr X and Chr Y carries an additional CO mark. (**B**) Variation of the number of MLH1 foci per pachytene nucleus of PWD and B6 males. The PAR-associated MLH1 foci were not counted. Each dot represents the MLH1 count of one pachytene spermatocyte. Vertical bars designate SD of individual males, horizontal bars represent the mean number of MLH1 foci per analyzed animal. Average MLH1 counts significantly differ between both strains.

As a proxy of meiotic CO rate we counted foci of the MLH1 mismatch repair protein in spermatocyte and oocyte pachytene spreads. Such approach has some limits; the number of MLH1 foci does not provide information about the genomic position of individual COs, so the observed differences in recombination rate cannot be allocated to particular chromosomes or genomic sites. Moreover, about 10% of COs might arise through an MLH1-MLH3-independent path and escape detection via MLH1 foci [15]. The pros of counting MLH1 foci lie in the direct quantification of actual CO events in individual cells and the relative ease of gathering a large amount of information on genome-wide CO rate in male and female genomes. The variation in global recombination rate between closely related (sub)species is genetically controlled [23,25]. In mice, the studied males of *Mmm* (sub)species showed higher meiotic CO rate than the males of *Mmd* and *Mmc* origin [24,25]. Consistent with these findings we observed significantly higher meiotic CO rate in *Mmm*-derived PWD males (mean 29.58, 95% confidence interval (CI) 28.66–30.56), than in meioses of the B6 strain, predominantly of *Mmd* origin (24.87, CI 24.29–25.47) (Fig 1).

### Autosomal modifiers of meiotic CO rate localize to Chr 7 and Chr 11

**Fig 2:**
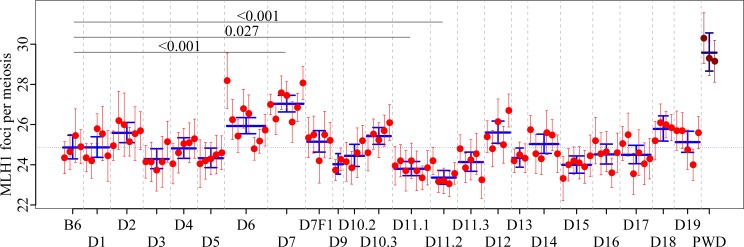
Meiotic recombination rate in consomic strains carrying PWD autosomes on B6 background. Each dot with error bars represents the mean number ± SEM of MLH1 foci per pachytene nucleus of one analyzed male. D1—D19 refers to B6.PWD-Chr# consomic strains carrying Chr 1^PWD^—Chr 19^PWD^ on B6 genetic background. The long blue bar with error bar represents the mean number of MLH1 foci and 95% CI per analyzed strain. The significance of the difference between B6 wildtype and a consomic strain is marked only when p<0.05.

To evaluate the role of individual PWD autosomes in the control of CO frequency we determined the mean number of MLH1 foci per pachynema in males of individual intersubspecific B6.PWD-Chr# chromosome substitution (consomic) strains and compared them with the parental B6 recipient strain or among themselves (Fig 2, S1 Table). Chromosome substitution strains fractionate phenotypic and genomic variation between the donor and the recipient inbred strain chromosome-wise, due to the fact that each strain of the panel carries one chromosome or its part from the donor strain, in our case PWD, on the genetic background of the recipient strain–B6 [32–34].

Altogether we examined 21 autosomal consomic strains for the male CO rate. Strains B6.PWD-Chr 8 and B6.PWD-Chr 10.1 were lost before the experiment had started; hence the effect of Chr 8^PWD^ and the proximal part of Chr 10^PWD^ could not be evaluated. The variance of MLH1 counts showed positive correlation with the average MLH1 counts at both intra-individual (Spearman´s *rho* = 0.20, p = 0.031) and intra-genotype levels (Spearman´s *rho* = 0.58, p = 0.003, S1 Fig). Comparison of individual consomic strains with the B6 parental strain revealed a significant increase of recombination rate in males carrying Chr 7^PWD^ (27.04, CI 26.63–27.46, p<0.001, Dunnet's post-hoc test, S2 Table). The PWD allele of genetic factor(s) on Chr 7 responsible for elevation of the recombination rate is recessive because five B6.PWD-Chr 7^PWD/B6^ heterozygotes displayed 25.15 (CI 24.63–25.70) foci per cell, a value not significantly different from the B6 parent (p = 1, S2 Table). The proximal and middle part of Chr 11^PWD^ were associated with a significantly lower mean count of MLH1 foci (23.79, CI 23.46–24.16, p = 0.027, and 23.36, CI 23.03–23.72, p = 0.001), than the mean counts of B6 parental strain, pointing to the transgressive effect of the underlying modifiers. At least two modifiers may be envisaged to control the suppressive effect of Chr 11^PWD^ on meiotic CO rate, the first one localized in the overlapping interval of PWD sequence in B6.PWD Chr 11.1 and B6.PWD Chr 11.2 (Chr 11, 44.0–75.6 Mb, GRCm38) and the second one in the 4.2 Mb interval of PWD sequence present in B6.PWD Chr 11.2 but absent in B6.PWD-Chr 11.1 and B6.PWD-Chr 11.3 males (Chr 11, 75.6–79.8 Mb, S2 Fig).

### *Prdm9* null mutant and *Prdm9* supernumerary copies do not change the meiotic recombination rate

**Fig 3:**
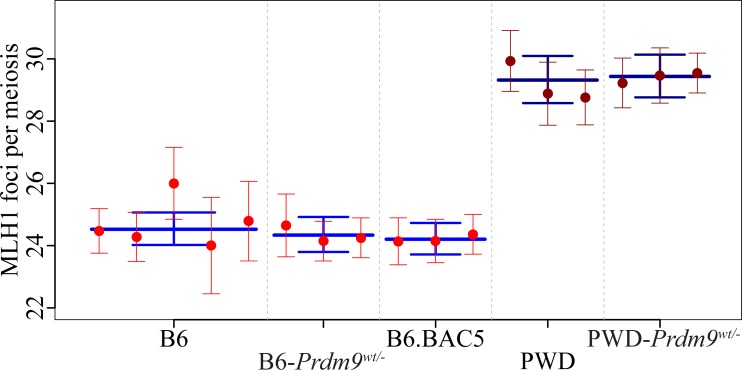
Meiotic recombination rate in males heterozygous for null mutation or carrying extra copies of *Prdm9*. The mean number of MLH1 foci depends on the genetic background of B6 and PWD males but does not reflect differences in the *Prdm9* copy number. See Fig 2 for legend.

*Prdm9* determines localization of hotspots of meiotic COs in most studied mammalian genomes [11] and shows a minor effect on the male recombination rate in humans [35]. Previous studies of two F2 populations of mouse strains, (PWD x CAST) [24] and (B6 x CAST) [26], reported no effect of Chr 17 carrying *Prdm9* on the global recombination rate. Since the change of *Prdm9* dosage partially rescues hybrid sterility of PWD x B6 F1 males [27,36], we analyzed the effect of a *Prdm9* null allele and of two extra transgenic copies of *Prdm9* on the meiotic CO rate (Fig 3, S3 Table).

The mean number of MLH1 foci per cell in four B6 males (24.52, CI 24.02–25.06) was not significantly different from knockout heterozygotes B6-*Prdm9*^*tm1Ymat/wt*^ [27] (24.34, CI 23.78–24.92) and from B6 males carrying two copies of the BAC5 transgene containing *Prdm9*^*C3H*^ (24.20, CI 23.71–24.73) [27,30]. After transferring the *Prdm9*^*tm1Yma*^ knockout to the PWD genetic background by 10 generations of backcrosses, *Prdm9*^*wt/-*^ heterozygotes displayed high counts of MLH1 foci, 29.61, CI 28.87–30.40 per cell, value not significantly different from that shown by PWD wildtype males (29.31, CI 28.58–30.10). To conclude, neither the extra *Prdm9* copies nor the deficiency of *Prdm9* in the PWD genome alters the global recombination rate when monitored by the mean number of MHL1 foci per meiosis, strongly indicating an independent control of global crossover rate variation and genomic crossover placement.

### X-linked hybrid sterility genomic locus harbors the strongest, male-specific modifier of global CO rate

**Fig 4:**
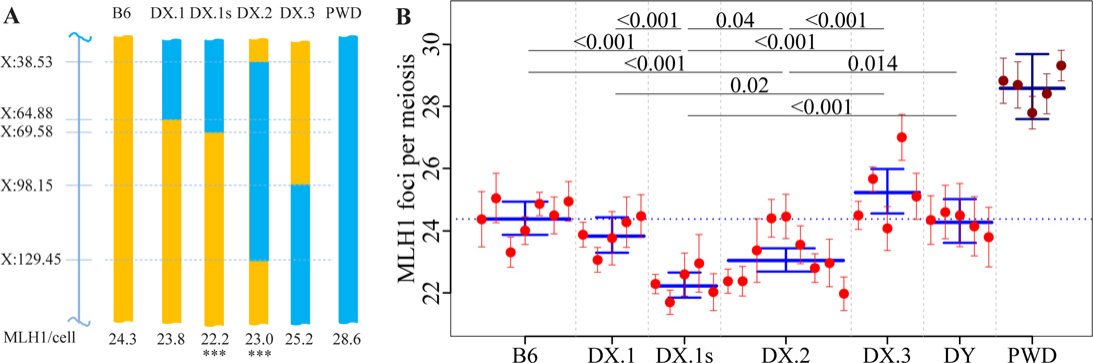
Fine mapping of meiotic recombination 1, *Meir1*, on Chr X^PWD^ using subconsomic strains. (**A**) The 4.7 Mb interval of the proximal part of Chr X shared by B6.PWD-Chr X.1s and B6.PWD-Chr X.2, but absent in B6.PWD-Chr X.1 and B6.PWD-Chr X.3, harbors a transgressive modifier of meiotic recombination rate *Meir1*. The Y axis shows the distance from the centromere in megabases (GRCm38). Chromosome intervals carrying the B6 DNA sequence are depicted in orange, the PWD sequence in blue. (**B**) The B6.PWD-Chr X.# subconsomics are abbreviated DX.# in column scatters. The significance of differences between strains (excluding PWD) is marked only when p<0.05. See Fig 2 for legend.

Previous studies have shown the fundamental impact of genetic factors located on mouse Chr X not only on the meiotic CO rate [24–26,29,37], but also on the inter-subspecific reproductive isolation [28,38–43]. In (B6 x CAST)F2 males, the strongest meiotic CO rate QTL was mapped on Chr X (25.4–42.4 cM, the highest LOD score Chr X:106.8 Mb) [26] and the analysis of the (PWD x CAST)F2 population revealed a strong QTL within the 65.5–84.5 Mb interval. In both studies the X-linked QTLs displayed transgressive effects, since the allele derived from the high CO rate parent acted in the opposite direction and caused decreased recombination frequency in the F2 hybrids [24]. We characterized the effect of Chr X^PWD^ on meiotic CO rate using the same four B6.PWD-Chr X # subconsomic strains that we developed for mapping the *Hstx2* hybrid sterility gene [28] (Fig 4, S4 and S5 Tables).

The B6.PWD-Chr X.1 strain, which carries 64.9 Mb of the PWD sequence starting from the centromeric end of Chr X^PWD^ displayed 23.74 (CI 23.30–24.44) MLH1 foci per cell, a value not significantly different from the B6 parent (24.40, CI 23.87–24.94, p = 0.71, Tukey's post-hoc test, S5 Table). Also B6.PWD-Chr X.3 males with the PWD sequence at the telomeric end did not differ from B6 (25.34, CI 24.56–25.98, p = 0.357). However, B6.PWD-Chr X.1s (69.6 Mb of proximal PWD sequence) and B6.PWD-Chr X.2 displayed a significantly lower number of MLH1 foci, 22.16 (CI 21.85–22.66) and 22.96 (CI 22.69–23.44), respectively (p<0.001). We designated the X-linked genetic factor present in the 4.7 Mb interval in B6.PWD-Chr X.1s and B6.PWD-Chr X.2 but absent in B6.PWD-Chr X.1 as Meiotic recombination 1, *Meir1*. The same 4.7 Mb locus (X:64,880,641–69,584,093, GRCmm38, see S1 Table in [28]) harbors hybrid sterility genes *Hstx1* and *Hstx2* [28,44]. Interestingly, the transgressive effect of the *Meir1*^*PWD*^ allele coming from the parent with high recombination rate but acting in the opposite way on B6 background parallels the effect of *Hstx2*^*PWD*^, which causes small testis size and lack of sperm in (PWD x B6)F1 hybrids [45]. Moreover, *Hstx1*^*PWD*^ causes sperm malformations on the B6 background [44], a phenotype not seen in either parent.

### X-linked control of female meiotic CO rate

**Fig 5:**
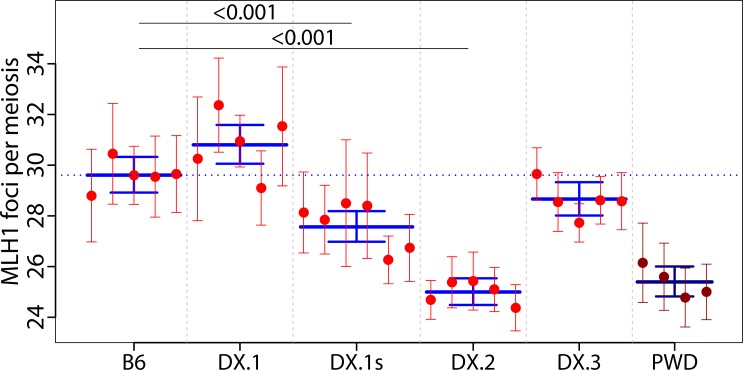
Mean MLH1 counts in female meiosis of Chr X subconsomic strains. The PWD genomic sequence present in B6.PWD-Chr X.2 carries the strongest female-specific modifier of meiotic recombination rate. For strain abbreviations see Fig 4. For detailed analysis of differences between individual subconsomics, see S7 Table. See Fig 2 for legend.

*Meir1* maps to the same genomic locus as hybrid sterility gene *Hstx2*, and both genetic factors operate in a transgressive mode, early at the first meiotic prophase. *Hstx2* interacts with *Prdm9* to control hybrid sterility of inter-subspecific hybrids in a male-specific manner [28,45]. These facts and the divergent modes of global CO rate regulation in males and females [24,37] led us to investigate the possible activity of *Meir1* in female meiosis. We estimated the mean MLH1 counts in pachytene oocytes from 18.5–19.5 dpc female fetuses of four B6.PWD-Chr X.# strains and PWD and B6 parents (Fig 5, S6 and S7 Tables).

In agreement with previous findings [24], B6 females displayed an average 4.7 MLH foci per cell more than males (29.61, CI 28.92–30.33 vs 24.87, CI 24.29–25.47), but the opposite effect was seen in PWD mice showing higher CO rate in males (29.58, CI 28.66–30.56 MLH1 foci per meiosis) than in females (25.40, CI 24.82–26.00). The MLH1 frequency in B6.PWD-Chr X.1s females showed significant depression compared to B6 females (27.56, CI 26.98–28.19 vs 29.61, CI 28.92–30.33, p<0.001 Tukey's post-hoc test, see Fig 5 and S7 Table). However, in contrast to the transgressive effect of *Meir1* in males, the change in B6.PWD-Chr X.1s females was in the same-sense direction. These findings support the male-specific activity of *Meir1*. PWD sequence X:69.58 Mb—98.15 Mb in B6.PWD-Chr X.2 subconsomic females caused the strongest reduction of meiotic CO rate (25.00, CI 24.49–25.54) to the level seen in PWD females, pointing to the presence of one or more major female-specific modifiers. Contrary to the transgressive effect of male X-linked modifiers ([24,26] and this study), this female-specific factor acted in accord with its PWD origin by suppressing the global CO rate.

### Frequency of zygonema DNA double-strand breaks in mice with distinct meiotic recombination rates

**Fig 6:**
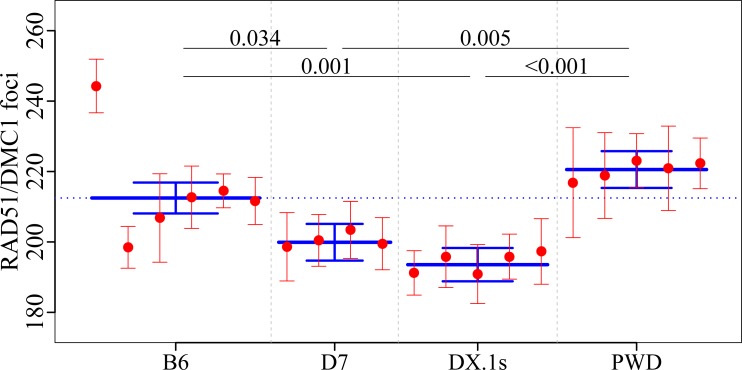
Mean RAD51/DMC1 counts in males with distinct meiotic recombination rates. The average frequency of DSBs in B6.PWD-Chr 7 and B6.PWD-Chr X.1 is lower than in both parental strains. For detailed analysis of differences between individual subconsomics, see S9 and S10 Tables.

Recent intraspecific comparisons of the crossover rate at pachytene stage of meiosis indicated direct proportionality of MLH1 foci variation to the DNA DSB frequency [3,46]. Nevertheless, a simple formula for direct conversion of DSB frequency into CO rate seems unlikely because the *Spo11* gene dosage variation does not affect the MLH1 counts, although it changes the DSB frequency, a phenomenon known as homeostatic control of recombination [14]. Comparison of B6 and PWD mid-zygonemas did not show a significant difference in the frequencies of Rad51/DMC1 foci (212.48, CI 208.09–216.87 vs 220.56, CI 215.33–225.78, p = 0.227, robust linear mixed model, Fig 6, S9 and S10 Tables).

However, one B6 male was an outlier with high RAD51/DMC1 counts. Accidentally, this animal was from a different breeding room than the remaining five males. Excluding the outlier, the PWD zygonemas would show significantly higher counts (p< 0.001) of cytologically detected DSBs than those of B6 origin. However, regardless of whether the B6 outlier is included or not, the two consomics, namely B6.PWD-Chr 7, showing a significantly enhanced rate of MLH1 foci, and B6.PWD-Chr X.1s, showing a strong depression of meiotic recombination rate, displayed significant decrease of RAD51/DMC1 counts (Fig 6, S9 and S10 Tables). Thus the inter-subspecific comparisons do not support direct proportionality between the DSB frequency and the meiotic recombination rate and may indicate uncoupling of DSB programming from global crossover rate control in inter-subspecific hybrids.

## Discussion

This study compares the global meiotic recombination rate between two closely related subspecies of the house mouse, *Mmm* represented by the PWD inbred strain and *Mmd* substituted by the B6 laboratory inbred strain. Previously, both strains served to model inter-subspecific F1 hybrid sterility characterized by disturbed synapsis of homologs, transcriptional dysregulation of the X chromosome and late pachytene arrest [28,45]. The infertility of PWD x B6 F1 hybrid males is under the control of epistatic interaction between the *Prdm9* gene on Chr 17 and X-linked *Hstx2* genomic locus [39]. Since *Prdm9* controls not only hybrid sterility but also fine-scale localization of CO hotspots [8,9,47], and because Chrs X of different mouse subspecies exerts a strong influence on the global CO rate [24,25,37], we decided to seek a possible link between meiotic recombination and hybrid sterility. Here we evaluated the effect of individual PWD chromosomes on genome-wide recombination rate in the B6 genome by means of B6.PWD-Chr # chromosome substitution strains [32]. The chromosome substitution strains partition the variation between two parental strains and provide a permanent resource allowing gradual accumulation of information on any heritable trait, including global recombination rate [34,48].

### Control of CO rate by PWD chromosomes in male meiosis

Analysis of MLH1 profiles of PWD and B6 parental strains revealed an increase of 4.7 MLH1 foci per cell in PWD male meiosis. Individual chromosome substitution strains showed a significant effect of PWD chromosomes in the case of Chr 7^PWD^ (+2.17 MLH1 foci per cell), middle region of Chr 11^PWD^ (-1.51 foci) and *Hstx2* genomic locus of Chr X^PWD^ (-2.24 foci). Information on Chr 8^PWD^ and the proximal part of Chr 10^PWD^ is missing because the strains carrying them were not available. In humans and mice, the variation in CO rates is associated with SUMO ligase RNF212 and ubiquitin ligase HEI10 [20,35,49]; however, the mouse Chrs 5 and 14, which carry their orthologs, showed no effect. The effect of the *PRDM9* gene on human genome-wide CO rate [35] and global recombination rate in cattle [21] was not paralleled by a change of CO rate in B6.PWD-Chr17 males. Since the copy-number variation of *Prdm9* gene alters hybrid sterility, we evaluated its effect on MLH1 rate. Two additional copies of functional *Prdm9* had no effect, but more remarkably, PWD males homozygous for *Prdm9* deletion kept the same high frequency of MLH1 foci as shown by PWD wild-type or hemizygous males. Recently, a decrease by about 11% DSBs determined as RAD51 foci in late leptonema was found in PRDM9-deficient versus PRDM9^B6^- carrying males on *Mmd*^*B6*^ background [50]. We can conclude that, at least on the *Mmm*^*PWD*^ genetic background, the functional PRDM9 protein is not essential for meiotic recombination and setting up the appropriate level of meiotic CO rate.

Two genetic studies on the meiotic CO rate showed partially overlapping autosomal QTLs in *Mmm*, *Mmd* and *Mmc* subspecies [24,26]. The strongest QTLs in (*Mmc* x *Mmm*)F2 population were revealed on Chr 7 and Chr X, the same two chromosomes involved in the CO rate control of *Mmm/Mmd* in the present study. Three weaker QTLs were mapped on Chr 3, Chr 15 and Chr 17 in both F2 crosses and three others were specific for a particular cross. We can conclude that the variation of the meiotic recombination rate between representatives of three mouse subspecies is controlled by a discrete number of genetic loci, some of which may be shared in different subspecies combinations. The Chr X harbored the strongest modifiers with transgressive effect in all three inter-subspecific comparisons.

### Male-specific meiotic recombination 1 (*Meir1*) maps within the hybrid sterility X 2 (*Hstx2*) genomic locus

We localized the underdominant (transgressive) *Meir1* into the 4.7 Mb interval on Chr X (X:64,880,641–69,584,093) previously shown to carry hybrid sterility *Hstx1* and *Hstx2* genetic factors [28,44]. *Hstx1* controls reduced fertility due to abnormal spermiogenesis in B6.PWD-Chr X.1s males, as independently confirmed by backcross mapping [44]. The *Hstx2*^*PWD*^ allele is associated with intrameiotic arrest and full sterility in PWD x B6 F1 hybrid males [28]. (B6.PWD-Chr X.1s x PWD)F1 males are completely sterile in contrast to (B6.PWD-Chr X.1 x B6)F1 males equipped with the *Hstx2*^*B6*^ allele [28]. All three genetic factors act in spermatogenesis in a sex-specific mode, and show transgressive effects on their respective phenotypes in inter-subspecific interactions. Such coincidence is remarkable but could have a simple explanation if all three phenotypes posed the pleiotropic effects of the same gene. The ultimate solution will require completion of positional cloning of these three factors and identification of appropriate candidate genes. There are six known protein-coding genes expressed in spermatogenesis in the 4.7 Mb interval; however, none of them has a known DNA DSB repair or other meiotic recombination function. *1700030B21Rik* and *4933436I01Rik* show only postmeiotic expression and the remaining four (*Slitrk2*, *Fmr1*, *Fmr1nb and Aff2*) are transcribed at early meiotic prophase I [51], thus being more likely candidates. The interval also harbors RNA genes including a cluster of miRNA genes, some of which show differences in meiotic expression between PWD and B6 males [28]. Uncovering the candidate gene for *Meir1* and evaluation of its relation to *Hstx1* and *Hstx2* could be facilitated if the candidate region could be shortened by recombination. Our attempts to further genetically dissect the 4.7 Mb genomic locus have so far been unsuccessful. The suspicion that the *Hstx2* locus lies in a recombination coldspot has strengthened after inspecting the same interval in recombinant inbred lines of Collaborative Cross, (http://csbio.unc.edu/CCstatus/index.py?run=CCV). In contrast with adjacent sites on Chr X, the *Hstx2* interval (62.1–66.8 Mb, GRCm37) carried no crossovers in 71 CC lines (S3 Fig) constructed from eight founder mouse strains by 20 generations of brother-sister inbreeding [52,53].

Dumont and Payseur [24] mapped the strongest QTL for F2 variation in the MLH1 foci count in crosses of PWD (*Mmm*) and CAST (*Mmc*) to the 68.5 Mb—87.3 Mb (95% CI, GRCm38) region overlapping the *Meir1* candidate region reported here. The highest LOD score was also exhibited by the QTL on Chr X in the F2 population of CAST and B6 inbred strains and again, the QTL from higher recombination B6 strain was associated with low recombination rate in F2 males [26]. This QTL was mapped more distally, the peak location being 55.6 cM or 106.8 Mb. Of interest in this context may be the recent finding of the effect of *Tex11* dosage, the meiosis-specific gene situated at 100.8 Mb on mouse Chr X, which significantly affected the number of MLH1 foci in both sexes [54]. The possible role of Chr X in the control of recombination rate variation was shown in a series of inter-subspecific reciprocal crosses of inbred strains derived from *Mmm* (PWD, CZECHI), *Mmd* (WSB, PERA) and *Mmc* (CAST, CIM). In all combinations tested the X chromosome from a low recombination strain was associated with higher recombination in F1 hybrids and vice versa [25]. Similarly, analysis of G1 generation males from the Collaborative Cross project revealed that CAST Chr X (coming from the low recombination rate strain) is associated with expansion of the male genetic map, while the *Mmm* Chr X^PWK^ is associated with contraction of the map, and the *Mmd* Chr X yields intermediate results [37].

### Sex-specific control of meiotic recombination

The genetic mapping studies of mice and humans show that beside recombination hotspots common for male and female meiosis, evidence is available for male- and female-specific hotspots or for sex-specific quantitative differences in the hotspot activity (citace). At the chromosomal level, the male-specific subtelomeric enhancement of CO frequency has been described in mice and humans [37,46,55,56]. Considering the global recombination level, the genetic length of the female genome is generally higher than that of males. The comparison of reciprocal F1 hybrids between mouse subspecies indicated the major role of the X chromosome in global recombination rate in males [25]. Here we mapped the transgressive genetic factor *Meir1*, responsible for *Mmm—Mmd* intersubspecific variation, to a 4.7 Mb genomic locus and showed its male-specific effect. The analysis of female meiosis revealed a major female-specific genetic factor located on Chr X distally from *Meir1* and acting in opposite, same-sense, direction. Testis-expressed gene 11, *Tex11* (X:100.8 Mb) [54], considerably modifies the recombination rate in both sexes depending on its copy number, but is situated 2.7 Mb outside of the border of PWD sequence in the B6.PWD- Chr X.3 subconsomic (X:98.1 Mb). The transgressive effect of *Meir1* and its mapping to the same 4.7 Mb genomic interval as hybrid sterility *Hstx2* provides indirect evidence in favor of the identity of both loci. However, no direct evidence is yet available in favor or against the hypothesis that the same genetic factor controls the recombination rate and hybrid male sterility.

To conclude, several observations link hybrid sterility to meiotic recombination. *Prdm9* histone H3K4 trimethyltransferase determines localization of recombination hotspots and simultaneously functions as a major hybrid sterility gene. *Meir1* controls the meiotic recombination rate and is localized in the same 4.7 Mb interval as *Hstx2*, the second major hybrid sterility gene. Both, *Meir1* and *Hstx2*, act in an underdominant manner on F1 hybrid background and both are sex-specific.

The intimate relation between meiotic recombination and reproductive isolation was reported in yeast inter- and intra-specific hybrids, where an overall genomic sequence divergence and the anti-recombination action of the mismatch repair system was shown to account for hybrid sterility [57–59]. In the mouse, variation in the genome-wide recombination rate was shown to initiate at the onset of the first meiotic prophase [3] The zygotene/pachytene stage of prophase I is the time point when the meiosis fails in PWD x B6 F1 hybrids, showing many homologs unsynapsed and spermatocytes predestined to apoptosis. Mapping the *Meir1* gene to a narrow candidate interval on Chr X is an important first step towards its positional cloning and elucidation of its relevance for reproductive isolation between closely related mouse subspecies.

## Materials and Methods

### Animals

Mice of the C57BL/6J inbred strain and *Mus m*.*m*.*-*derived PWD/Ph inbred strain, together with C57BL/6J-Chr #^PWD^ chromosome substitution (consomic) and subconsomic strains [32], were maintained in the Specific Pathogen-Free Facility of the Institute of Molecular Genetics in Prague. Male mice were sacrificed by cervical dislocation and whole testes were dissected at the age of 13–18 weeks for the MLH1 and RAD51/DMC1 immunostaining. Ovaries were removed from 18–19 days old female embryos.

### Ethics statement

The mice were maintained in accordance to animal care protocols approved by the Committee on the Ethics of Animal Experiments of the Institute (No. 141/2012). The animal care obeyed the Czech Republic Act for Experimental Work with Animals (Decree No. 207/2004 Sb and Acts Nos. 246/92 Sb and 77/2004 Sb), fully compatible with the corresponding regulations and standards of European Union (Council Directive 86/609/EEC and Appendix A of the Council of Europe Convention ETS123). The project number is 141/2012. The head of the Committee for Animal Wefare and protection is MVDr. Jan Honetschläger, MBA.

### Immunostaining, microscopy and data evaluation

Spreads of meiocytes were prepared as described [60] with modifications, and immunostained with the following primary antibodies; rabbit anti-SYCP1 (Abcam, # ab15087) diluted 1:500, mouse anti-MLH1 (Abcam, # ab14206) diluted 1:20, human anti-centromere protein (AB-Incorporated #15–235) diluted 1:300, rabbit anti-RAD51 (Santa Cruz, SC-8349) diluted 1:300, rabbit anti-DMC1 (Santa Cruz, SC-22768) diluted 1:300. Secondary antibodies: anti-Rabbit IgG AlexaFluor 488 (Molecular Probes, # A-11034) diluted 1:400, anti-Mouse IgG AlexaFluor 568 (Molecular Probes, # A-11031) diluted 1:400, anti-Human IgG AlexaFluor 647 (Molecular Probes, # A-21445) diluted 1:400. After adding of primary antibodies in 100 μl of MAH buffer (1.5% BSA, 5% goat serum, 0.05% Triton X-100 in PBS, 0,2x cocktail of protease inhibitors) slides were incubated overnight in a cold room covered with a cover glass and washed 3x in PBS 10 minutes each. Secondary antibodies in MAH buffer were applied and slides were incubated under a cover glass for 1 hour in a cold room in the dark. Slides were washed 3x in PBS 10 min each, air dried for 15 min in the dark at room temperature and mounted in Vectashield medium with DAPI.

Images were observed in a Nikon Eclipse 400 epifluorescence microscope equipped with single-band pass filters for excitation and emission of infrared, red, blue, and green fluorescence (Chroma Technologies) and Plan Fluor Objective 60x (MRH00601; Nikon). The images were captured with a DS-QiMc mono-chrome CCD camera (Nikon) and NIS-Elements imaging software. Pictures were adjusted for evaluation and foci were counted using the NIS-Elements program.

For counting MLH1 foci only autosomal MLH1 foci were scored in mid- to late pachynemas. To determine the RAD51/DMC1 foci count, we scored zygotene spermatocytes (predominantly in mid-zygonema) with bright signal of SCP3 and RAD51/DMC1. RAD51 and DMC1 proteins were labeled with the same secondary antibody and counted together. Altogether 4–5 animals per strain and 20 or more cells per mouse were evaluated for both MLH1 and RAD51/DMC1.

### Statistics

All calculations were performed in the statistical environment R 3.2.2 [61] and using packages lme4 [62], robustlmm [63] and multcomp [64,65]. The MLH1 counts were not normally distributed, exhibited heteroscedasticity and were typically clustered for individual mice. Moreover, the numbers of evaluated cells were mostly 20, but for a few individuals they were much higher. All these features of the data were properly modeled by generalized linear mixed model (GLMM) or generalized linear model (GLM). The data analyzed using GLMM with Poisson error distribution with a log-link function were fitted using restricted maximum likelihood (REML) and Gauss-Hermite quadrature with 20 quadrature points. The logarithm of the intensity parameter was fitted as dependent on the genotype (fixed effect) and individual mouse (random effect nested within genotype fixed effect) plus 19 because every cell has at least 19 foci,

$$Y_{ij}∼Po\left(\lambda_{ij}\right),\;\text{log}\left(\lambda_{ij}\right)=19+\alpha \left({geno}_{i}\right)+\beta ({mouse}_{ij})$$

Since the *β(mouse)* effect was missing for *Prdm9* copy number variation data the GLM was fitted instead. All consomic and subconsomic strain effects were compared to B6 control group. The individual members within the sets of subconsomic strains for Chr 11 and Chr X were compared to each other. The data from mouse strains differing in the copy number of *Prdm9* were compared to each other with Tukey’s method as a multiple-testing procedure. RAD51/DMC1 counts were analyzed using the robust linear mixed model [60] with the mean dependent on the genotype (fixed effect) and individual mouse (random effect nested within genotype fixed effect).

$$Y_{ijk}=\alpha \left({\mathrm{geno}}_{i}\right)+\beta ({mouse}_{ij})+\;\varepsilon_{ijk},\;\varepsilon_{ijk}∼N(0,\sigma^{2})$$

The Bonferonni correction was applied to the set of comparisons: B6 to PWD and B6 and PWD to other genotypes–to achieve family-wise error rate of 0.05.

## Supporting Information

S1 Fig Correlation of mean and variance in autosomal chromosome substitution strains. Comparison of MLH1 average vs variance calculated at the individual level (left) and strain level (right). There is a positive correlation between averages and variances both for individuals (Spearman’s ρ = 0.20, p = 0.031) and strains (Spearman’s ρ = 0.58, p = 0.003).
(EPS)

S2 Fig Impact of Chr 11 subconsomics on meiotic recombination rate. Scheme of overlapping intervals of the PWD sequence on Chr 11 among B6.PWD-Chr 11.1, B6.PWD-Chr 11.2 and B6.PWD-Chr 11.3 subconsomics. The borders of PWD intervals were mapped to the closest SNP marker in megabase scale (GRCm38) Asterisks depict possible localization of meiotic CO modifiers.
(TIF)

S3 Fig Crossover numbers in the 77 Collaborative Cross lines in the proximal area of Chr X divided in 5-Mb bins. The *Hstx2* interval (62-66Mb, GRCm37) behaves as a cold spot of recombination.
(PNG)

S1 Table Summary of MLH1 counts in chromosome substitution strains with PWD autosomes. (XLSX)

S2 Table Statistics for MLH1 counts in chromosome substitution strains with PWD autosomes. (XLSX)

S3 Table Summary of MLH1 counts in Prdm9 knockout and transgenic males. (XLSX)

S4 Table Statistics for MLH1 counts in Prdm9 knockout and transgenic males. (XLSX)

S5 Table Summary of MLH1 counts in sex chromosome substitution strains. (XLSX)

S6 Table Statistics for MLH1 counts in sex chromosome substitution strains. (XLSX)

S7 Table Summary of MLH1 counts in female X-chromosome substitution strains. (XLSX)

S8 Table Statistics for MLH1 counts in female X-chromosome substitution strains. (XLSX)

S9 Table Summary of RAD51/DMC1 counts. (XLSX)

S10 Table Statistics of RAD51/DMC1 counts. (XLSX)
