# Supplementary figures and images for: Hybrid Sterility Locus on Chromosome X Controls Meiotic Recombination Rate in Mouse
Source: PLoS Genet. 2016 Apr 22;12(4):e1005906. doi: 10.1371/journal.pgen.1005906 (PMC4841592; doi:10.1371/journal.pgen.1005906)

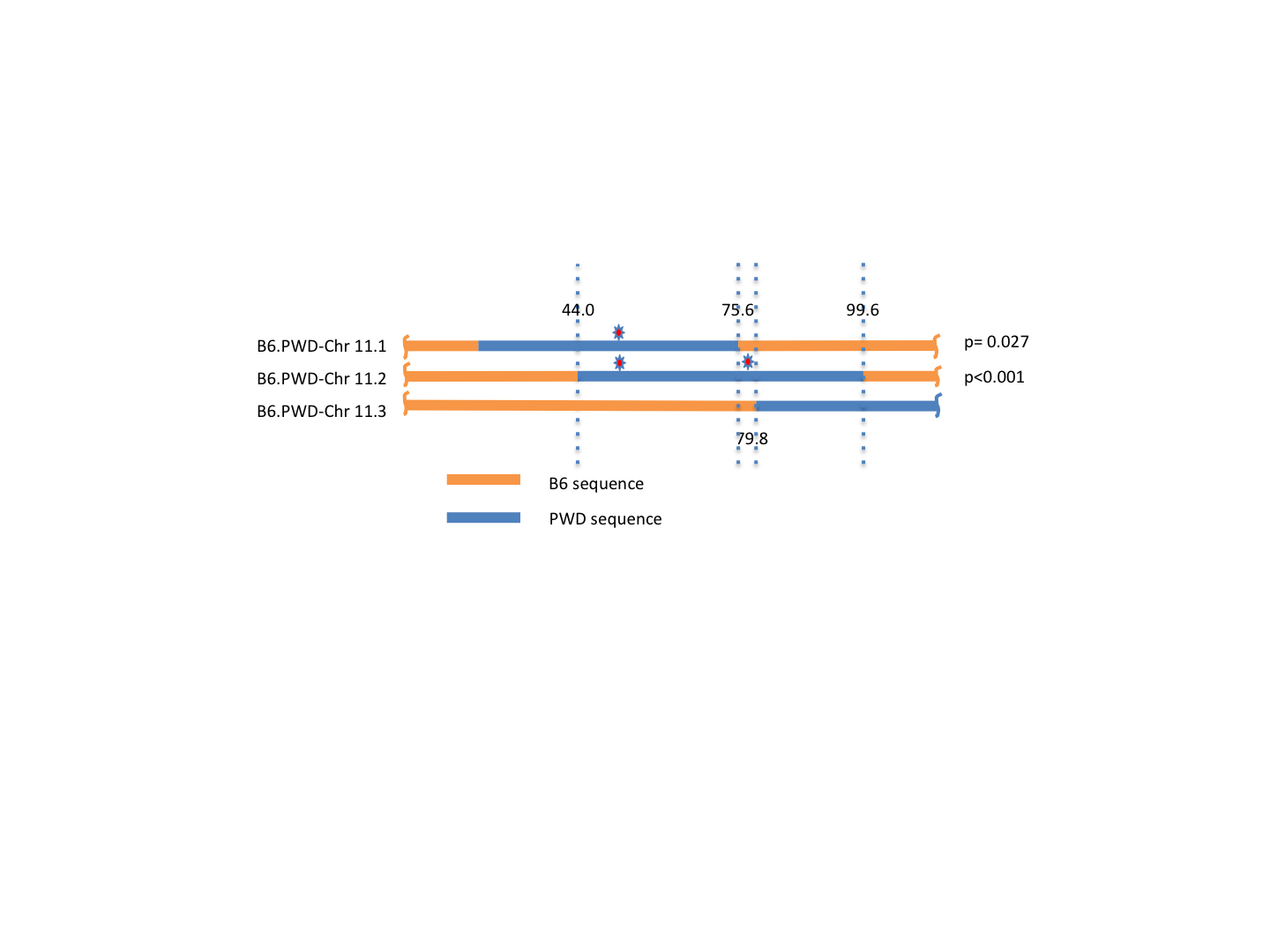

Supplement: S2 Fig — Scheme of overlapping intervals of the PWD sequence on Chr 11 among B6.PWD-Chr 11.1, B6.PWD-Chr 11.2 and B6.PWD-Chr 11.3 subconsomics. The borders of PWD intervals were mapped to the closest SNP marker in megabase scale (GRCm38) Asterisks depict possible localization of meiotic CO modifiers. (TIF) [file pgen.1005906.s002.tif]

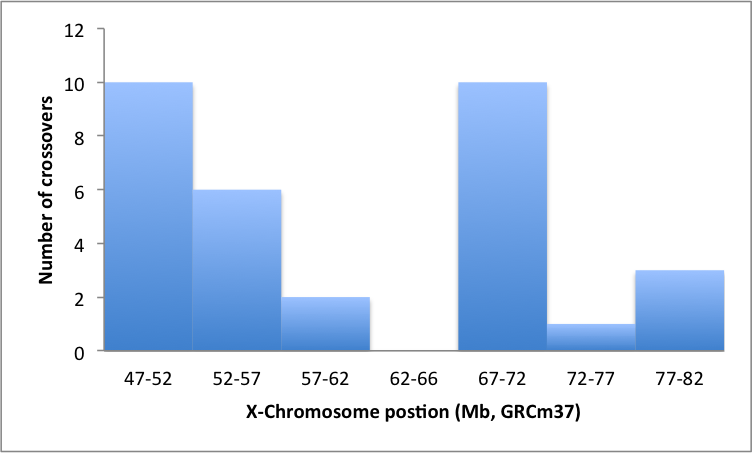

Supplement: S3 Fig — The Hstx2 interval (62-66Mb, GRCm37) behaves as a cold spot of recombination. (PNG) [file pgen.1005906.s003.png]
